# geneCommittee: a web-based tool for extensively testing the discriminatory power of biologically relevant gene sets in microarray data classification

**DOI:** 10.1186/1471-2105-15-31

**Published:** 2014-01-30

**Authors:** Miguel Reboiro-Jato, Joel P Arrais, José Luis Oliveira, Florentino Fdez-Riverola

**Affiliations:** 1 Escuela Superior de Ingeniería Informática, Universidade de Vigo, Campus Universitario As Lagoas s/n, 32004 Ourense, Spain; 2 DETI/IEETA, University of Aveiro, 3810-193 Aveiro, Portugal; 3 Department of Informatics Engineering (DEI), Centre for Informatics and Systems of the University of Coimbra (CISUC), University of Coimbra, Coimbra, Portugal

## Abstract

**Background:**

The diagnosis and prognosis of several diseases can be shortened through the use of different large-scale genome experiments. In this context, microarrays can generate expression data for a huge set of genes. However, to obtain solid statistical evidence from the resulting data, it is necessary to train and to validate many classification techniques in order to find the best discriminative method. This is a time-consuming process that normally depends on intricate statistical tools.

**Results:**

geneCommittee is a web-based interactive tool for routinely evaluating the discriminative classification power of custom hypothesis in the form of biologically relevant gene sets. While the user can work with different gene set collections and several microarray data files to configure specific classification experiments, the tool is able to run several tests in parallel. Provided with a straightforward and intuitive interface, geneCommittee is able to render valuable information for diagnostic analyses and clinical management decisions based on systematically evaluating custom hypothesis over different data sets using complementary classifiers, a key aspect in clinical research.

**Conclusions:**

geneCommittee allows the enrichment of microarrays raw data with gene functional annotations, producing integrated datasets that simplify the construction of better discriminative hypothesis, and allows the creation of a set of complementary classifiers. The trained committees can then be used for clinical research and diagnosis. Full documentation including common use cases and guided analysis workflows is freely available at http://sing.ei.uvigo.es/GC/.

## Background

In recent years, there has been growing interest in incorporating expression microarrays as an effective technology for disease diagnosis and prognosis [1,2]. In this context, the identification and validation of specific gene expression signatures able to discriminate disease classes from microarray data represents a primary field in translational clinical research [3-5]. Consequently, several individual tools and integrative platforms have recently been developed [6-9], giving support to different functionalities related to (*i*) recognizing optimal gene sets for given data sets, (*ii*) performing functional analysis from a biological perspective and (*iii*) automatically building accurate classification models.

In this scenario, the M@CBETH [10] web server [11] implements a two-class microarray classification benchmarking tool able to find the best prediction among different classification methods (support vector machines and Fisher’s discriminant analysis) by using principal component analysis (PCA) to reduce the dimension of the data. Prophet [12] is another web-based tool [13] that automatically builds different classifiers (support vector machines, k-nearest neighbour, diagonal linear discriminant analysis, self-organizing maps and shrunken centroids) and allows them to be used for further sample classification following a leave-one-out cross-validation (LOOCV) schema. The web server supports two ways of ranking genes for subsequent selection (F-ratio and the Wilcoxon statistic) and includes the possibility of performing the functional interpretation of the molecular signature found. More recently, the FiGS [14] on-line workbench [15] builds up diverse gene selection procedures by aligning different feature selection techniques and classifiers. The workbench diversifies the gene selection procedures by incorporating gene clustering options in the feature selection step and different data pre-processing options in the classifier training step. All candidate gene selection procedures are evaluated through the .632+ bootstrap method.

From a different perspective, the Signature Evaluation Tool (SET) [16] provides a standalone application to assist in evaluation of the discrimination power of gene expression signatures. SET implements the Golub’s weighted voting algorithm and the LOOCV schema to distinguish between two supervised groups of samples.

Moreover, several ensemble strategies have also been proposed for microarray data classification [17-20] as a way to exploit multiple methods to discriminate the most relevant clusters from a set of genes. Although these methods are shown to be more robust for pattern detection in microarray data, one problem remains in their results: it is essential to find mechanistic explanations that allow understanding the reasons why a particular set of genes was under or up-regulated. Although the numerical analysis of data seems quite consolidated, its full integration with biological knowledge is still a long way off. In many cases, the signatures discovered by the data classification methods look more like random gene lists than biologically plausible and understandable patterns. In the same line, another shortcoming of standard classification algorithms is that they treat gene expression levels as anonymous attributes. However, a lot is already known about the function and role of many genes in certain biological processes. In this context, the inclusion of additional knowledge sources in the classification process can prevent discovery of the obvious, complement a data-inferred hypothesis with references to already proposed relations, avoid overconfident predictions and allow systematic relation of the analysis findings to present knowledge [21]. To cope with this situation, several authors have proposed the integration and use of external knowledge in ensemble-based predictors. Reboiro-Jato et al. presented genEnsemble [22] a novel classifier able to combine the advantages of ensemble approaches with the benefits obtained from the true integration of biological knowledge in the classification process. The authors demonstrate the robustness of the proposed method when applied to several microarray data in an inter-dataset scenario. Pang et al. combine a random forest (RF) classifier with pathway information to explore the capacity of RF to assess the importance of variables in deriving the enrichment of pathways [23]. In this sense, this work is more focused on the functional analysis than on the classification task. Functional analysis has also been the target for other researchers. FatiGO + [24] is a web-based tool specially oriented to the interpretation of microarray experiments. It uses concepts, such as gene ontology (GO), KEGG pathways, Interpro motifs, Swissprot keywords and text-mining based bioentities related to diseases and chemical compounds, to provide a comprehensive analysis of the results. GeneBrowser [25] is another web-based tool that aims to extract biological knowledge from a list of genes. It combines data from several sources and different visualisation methods to improve biological interpretation and knowledge extraction from a group of genes.

Each of these tools presents unique characteristics supporting specific tasks comprising the whole process of constructing and validating prediction rules based on genes. However, an easy-to-use tool assisting systematic evaluation and interpretation of the discriminant ability of biologically relevant gene sets (i.e.: genes involved in pathways, genes as target of microRNAs, genes in given locations, chemical interacting genes, genes related to syndromes, etc.) is still in high demand by clinical researchers. In this context, we present geneCommittee, an on-line web-based tool giving specific support for extensively testing the discriminatory power of biologically relevant genes in microarray data classification.

The main objective of our geneCommittee server is two-fold: to contribute to the systematic analysis of the discrimination power of genes that are mainly used by wet-lab researchers and practitioners; using available knowledge regarding genes and related biomedical concepts (gene locus, proteins, pathways, diseases, drugs, etc.) to enrich microarrays’ data features for more powerful discrimination of sets of genes. In order to accomplish these goals, custom experiments can be defined using several successful classifiers over different microarray data sets following several validation schemas. To our knowledge, there is no equivalent tool able to facilitate the systematic evaluation of biologically relevant gene sets following a straightforward classification perspective.

## Implementation

geneCommittee is implemented as an AJAX-enabled web application programmed in J2SE 1.6 Java language and it is designed to run on a standard Tomcat 6 Web application server. Its source code is publicly available in Github repository [26] being distributed under the GPL free software license. The ZK development framework [27] was used to construct a rich web user interface with many features of a desktop application.

### Architecture

**Figure 1:**
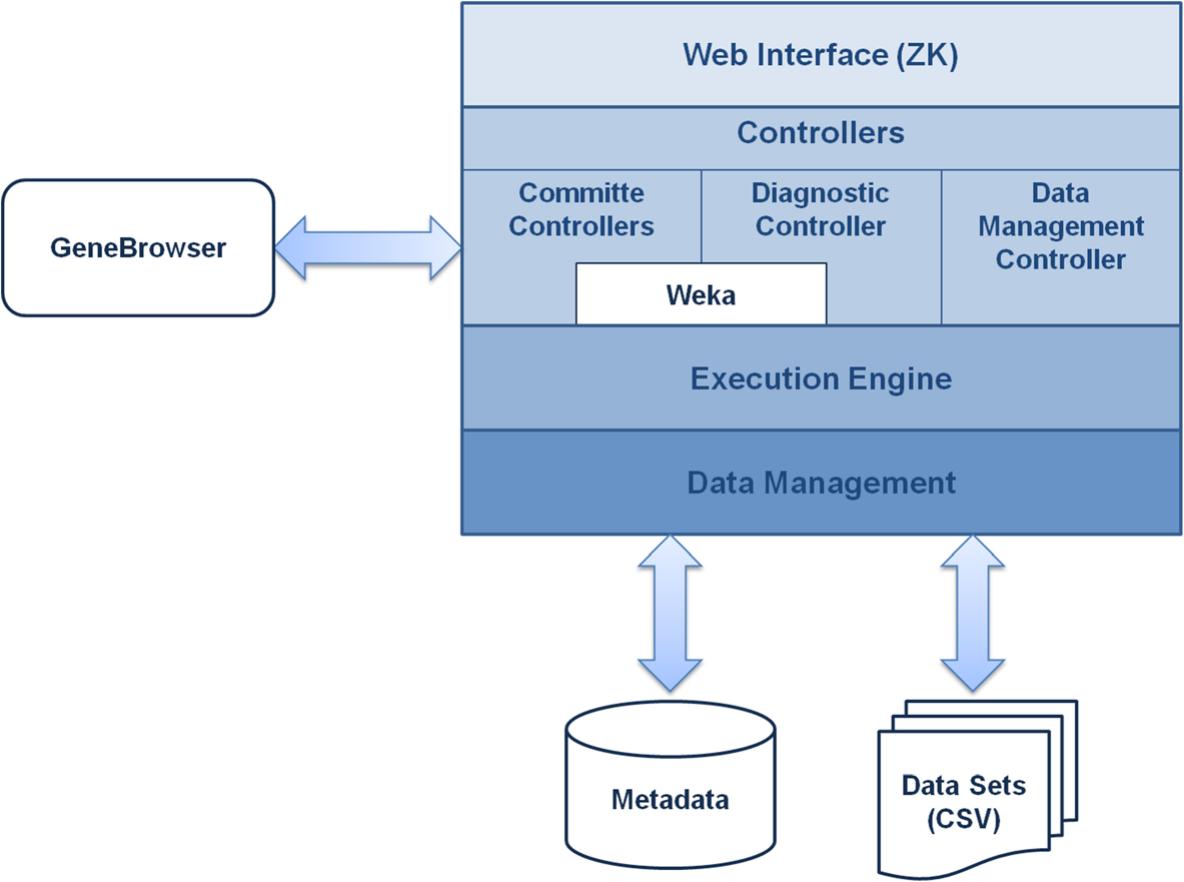
**General architecture of geneCommittee.** The architecture of geneCommittee integrates GeneBrowser and Weka applications and is divided into four main layers: (i) web interface, (ii) controllers, (iii) execution engine and (iv) data storage and management.

geneCommittee is based on a layered design in which the system was structured in four main tiers: (*i*) web interface, (*ii*) controllers, (*iii*) execution engine, and (*iv*) data management. Figure 1 shows a block diagram representing geneCommittee architecture and its relationship with other external entities.

As previously mentioned, the web interface uses the ZK framework, which eases the development of desktop-like web applications by providing a set of rich widgets together with an environment that uses intensive AJAX communications between the client and the server. In geneCommittee we have taken advantage of these features to provide a very intuitive user interface to guide the user through the main system workflow.

In the web interface, the application logic is managed by a set of specific controllers. The design of this layer is strongly based on the Model-View-ViewModel (MVVM) architectural pattern supported by the ZK framework, which reduces the coupling between the user interface and the controller classes. Depending on their primary role, geneCommittee defines three different types of controllers: (*i*) committee controllers, which manage the committee creation workflow, (*ii*) the diagnostic controller, which is responsible for the classification of new patients, and (*iii*) the data management controller, which is in charge of data set handling. Additionally, although not represented in Figure 1, other minor controllers handle secondary features like personal data modification, feedback report and help support. As showed in Figure 1, committee and diagnostic controllers use Weka [28] classifiers and feature selection algorithms to build the committees. Additionally, when training a new committee, a special controller will use the web service interface of the GeneBrowser system to enrich a list of previously selected genes.

The execution engine layer is probably the most important piece of geneCommittee, as it contains the core of the application. The execution engine runs almost every task asynchronously. This design principle provides us with two major advantages. On one hand, by having all the tasks running in one single class we can schedule their execution to avoid system overloads. Specifically, geneCommittee application allows the system administrator to select the maximum number of tasks that can be simultaneously executed as a way to control both memory and processor consumption. Moreover, the execution engine automatically interleaves the execution of different user tasks in order to avoid long waits. On the other hand, the execution engine is completely isolated from the user interface, being alive to complete the execution of pending tasks even when the user closes the session. In such a situation, users can request to receive an email when their tasks are finished.

At the bottom part of Figure 1, the data management layer is responsible for guaranteeing data persistence. geneCommittee stores training and test data sets in separate files while the remaining information (e.g. data sets metadata, user data, committees, etc.) is stored in a relational database. All the data is kept in the server at the time it is uploaded or generated, preventing data loss and stopping the user from making unnecessary save actions.

### Sources of available knowledge

In order to identify the biological discrimination power from the available classifiers, the gene sets need to be annotated with biomedical knowledge obtained from public repositories and databases. To address this challenge, geneCommittee applies a previously well-established and successful workflow supported by GeneBrowser application. All implemented services are based on a biomedical warehouse that has a generic database schema and supports an unlimited number of biological databases. Currently it includes data from public data sources, such as UniProt [29], Entrez gene [30], Gene Ontology, KEGG [31] and PubMed [32]. Overall it integrates 1000 species, representing over 7 million gene products with 70 million alternative gene/protein identifiers and 140 million associations with biological entities. Detailed information regarding the schema and the integrated databases is available in [33].

The programmatic access supported by GeneBrowser is used by geneCommittee to automatically annotate the list of genes with terms such as Gene Ontology terms, KEGG pathways and OMIM disease associations. This information will be used by the specialist to select meaningful features for training biological interpretable classifiers. In addition, these enriched groups can be further explored as datasets in GeneBrowser.

### System workflow

**Figure 2:**
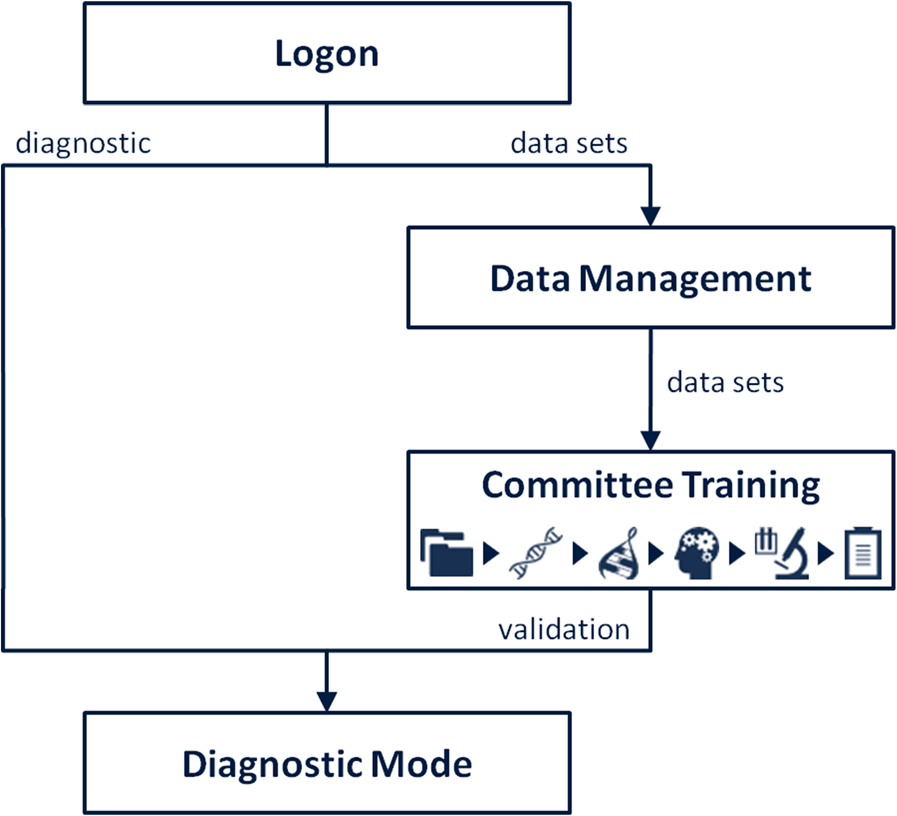
**geneCommittee workflow overview.** Users can train a new committee using a training dataset or they can reuse a previously defined committee to diagnose new patients.

With the goal of providing specific support to diagnostic analyses and related clinical management decisions, the workflow implemented in geneCommittee includes 4 distinct phases (see Figure 2): Access control, Data management, Committee training, and Diagnostic. This structure was planned with two different types of users in mind, one with a more statistical background, and another mainly interested in patients’ diagnostic. Nevertheless, any profile between both can easily take advantage of all the implemented functionalities.

In order to gain access to our tool, the first step is to create an account, which will allow each user to keep his own independent workspace. The demo account can also be used, providing a quick overview of the system functionalities. After login, two different paths can be independently followed. The first deals with the management of train datasets and the construction of classification models; the second is related with the exploitation of the developed models to identify specific patients in new datasets.

### Data management

In this area the user can upload and manage raw datasets that will be later used for training a specific committee. geneCommittee accepts comma separated value (CSV) files where samples are represented in columns and genes are placed in rows. Therefore, each sample (column) contains a *Sample ID* and a *Class*. Each gene (row) contains an *ID* and a *name*. Each cell in this sample/gene matrix specifies the expression value of a given gene corresponding to a specific sample. More detailed information about the format can be obtained in the user manual (see Additional file 1 for a more elaborated explanation). In addition to data import and export options, this area also allows the user to carefully search and inspect each uploaded dataset.

Datasets uploaded to geneCommittee should be previously normalized, as no pre-processing utilities are included. We recommend using the Robust Multichip Average (RMA) normalization technique [34].

### Committee training

**Figure 3:**
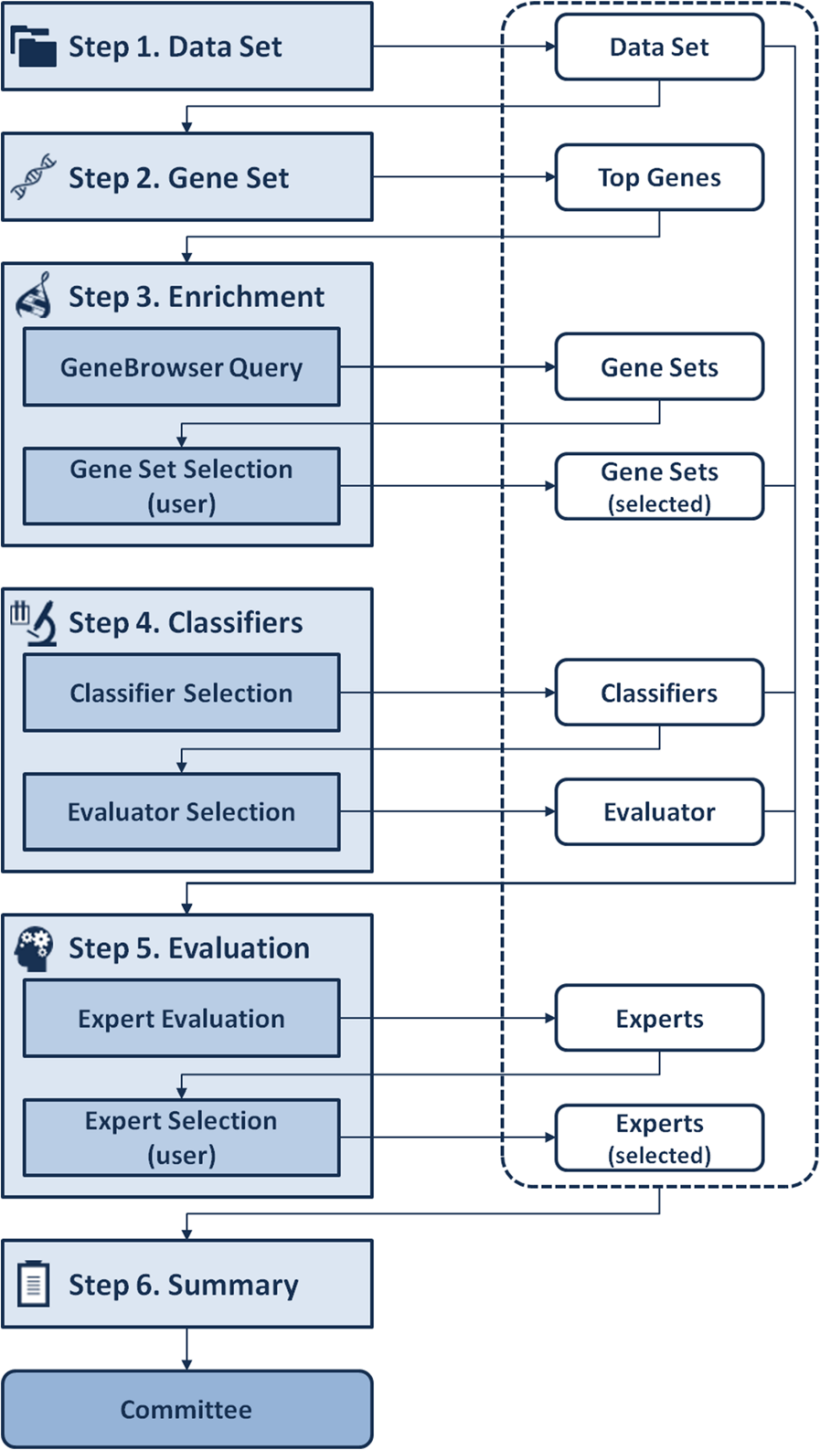
**Steps included in the Committee training wizard.** Left column shows the training steps and substeps while right column identifies the data generated during the training process.

The Committee training area is a key piece in the overall architecture of our geneCommittee server. It implements an easy-to-use and straightforward 6-step wizard for giving specific support to both (*i*) the initial enrichment of raw datasets and (*ii*) the later construction, training and validation of interpretable classifiers to finally build an accurate committee of *experts*. Figure 3 shows all the steps comprising the whole wizard together with the input and output entities stored by gene Committee.

In order to better understand the specific functionality of each block depicted in Figure 3, we briefly introduce every step comprising the Committee training wizard.

#### S1. Data set

The first action consists of selecting the desired dataset from those previously uploaded using the data management area. For each dataset, general information is shown regarding conditions and samples. Since the selected dataset will be subsequently used along the remaining pipeline, any change in this step will imply that the training process already performed with the actual dataset will be lost unless the whole workflow is completed and saved.

#### S2. Gene set

In this step the user can select those genes showing higher discriminative potential by using several well-established ranking methods: chi-squared distribution, information gain split method, gain ratio and the relief-f feature filtering algorithm. Additionally, numeric attributes can be converted to binary values and/or missing values merged in order to better adapt raw data to the preferred filtering algorithm.

#### S3. Enrichment

In this stage of the workflow previously selected genes are automatically enriched using GeneBrowser web services, which provide valuable biological knowledge regarding related enzymes, homologies, ontologies, proteins, pathways, diseases and drugs [25]. All this wealthy data gathered from multiple sources can be used to add new relevant features to be taken into consideration during the rest of the training process. As soon as the on-line gene enrichment process is finished, a new table is generated containing the name of the retrieved items, their sources, the associated p-value, the number of genes involved and a complementary link to GeneBrowser application. This link allows the user to obtain exhaustive information for entries of interest without leaving the geneCommittee workspace.

#### S4. Classifiers

After the gene set enrichment process, it is necessary to (*i*) specify and configure the classifiers that will be used and (*ii*) the desired global evaluation strategy. The global evaluation system implemented in geneCommittee is very flexible, allowing both the use of a single classification method or a specific combination of multiple classifiers. Since each classification algorithm has its own advantages and drawbacks, and taking also in consideration that its performance directly depends on the data, our proposal of having a committee of experts allows choosing the best combination of methods for a specific dataset. Regarding this feature, at the moment our geneCommittee server supports five different types of standard classifiers: (*i*) k-nearest neighbours, (*ii*) decision trees, (*iii*) support vector machines, (*iv*) naïve Bayes, and (*v*) random forest. According to the needs, the user can select any number of classifiers (belonging to the same type or not) for which each configuration can be individually established.

#### S5. Evaluation

**Figure 4:**
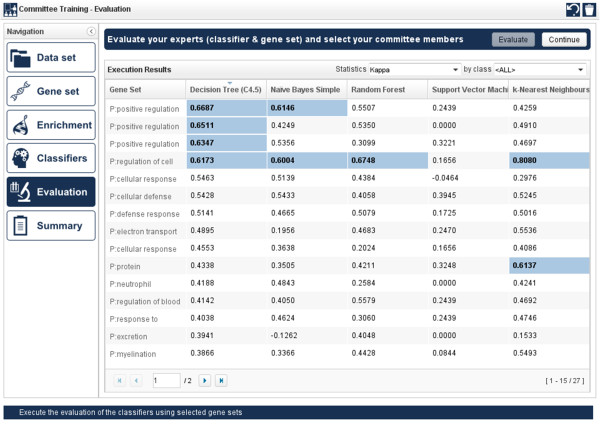
Selecting individual experts (classifier + gene set) for being members of a new committee.

Once the list of candidate classifiers is finally defined and the evaluation strategy is set, the evaluation step provides an interactive live visualization of the experiment execution. As soon as the job is finished, the user can choose the desired experts (combination of individual classifiers and gene features) to build a new committee (see Figure 4).

In order to evaluate the performance of each expert when dealing with the initially selected dataset, the statistical analysis carried out by geneCommittee can be conveniently adjusted. Implemented measures include Cohen’s Kappa, accuracy, precision, recall, specificity and F-measure, serving for a better perception of the results’ significance. Another interesting feature is the possibility of visualizing the results of a specific class, or in the case shown in Figure 4, a condition. Following the selection of those classifiers comprising the new generated committee, the user has to save it for further use to evaluate new samples (in Diagnostic mode).

#### S6. Summary

Finally, the last step included in the Committee training wizard is in charge of showing general information about the input dataset used for training, summarizing the criteria used for performing the gene selection process and introducing some details about the new available committee.

### Diagnostic mode

**Figure 5:**
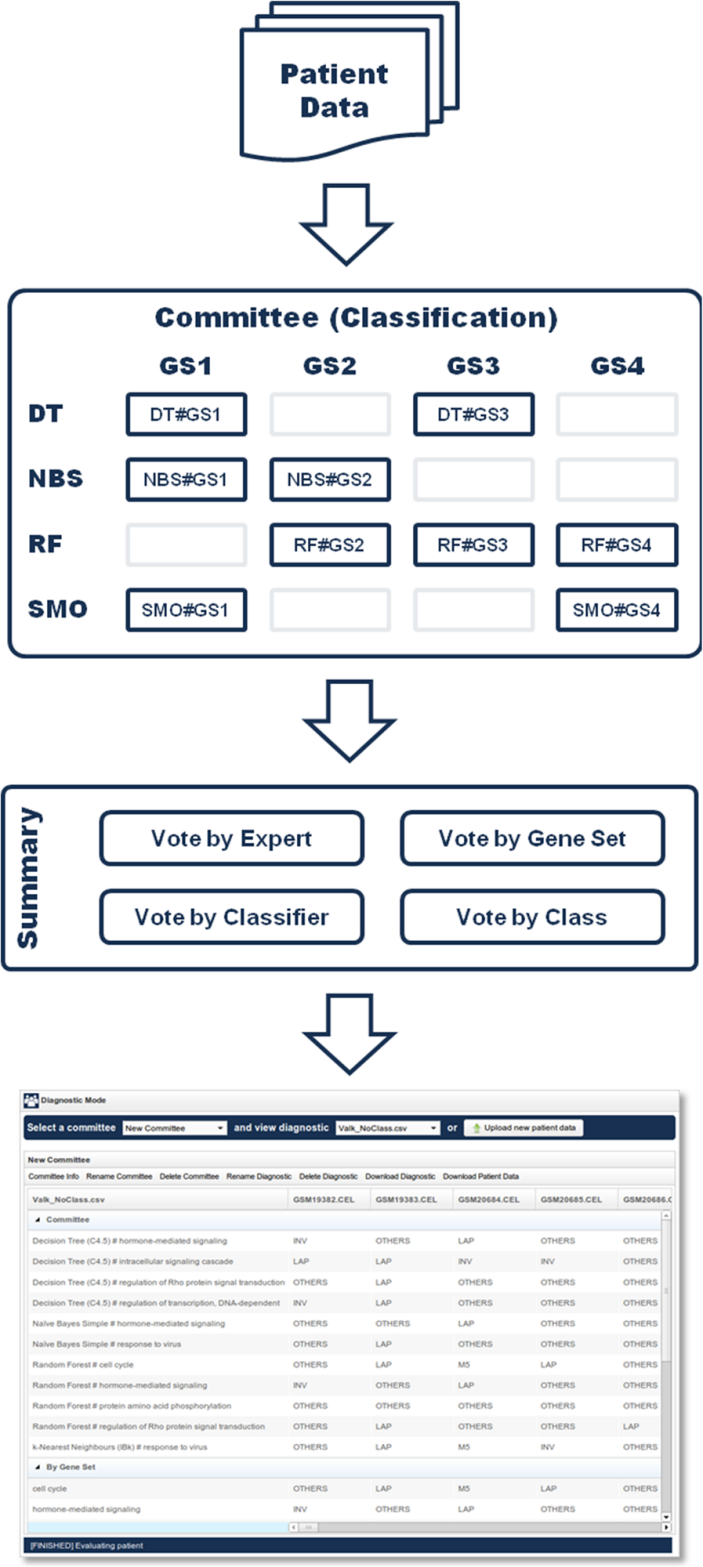
**Process carried out by geneCommittee working in Diagnostic mode.** The pipeline is divided into two main steps: (i) classification, where each selected expert classifiers new patients, and (ii) summary, where the classification results are summarized.

In this area it is possible to directly apply previously trained committees to evaluate new samples. As soon as the user uploads a new dataset corresponding to unseen patient data, the selected committee will start working on the diagnostic of all the samples to identify their corresponding classes (conditions). The whole process carried out by geneCommittee working in Diagnostic mode is showed in Figure 5.

**Figure 6:**
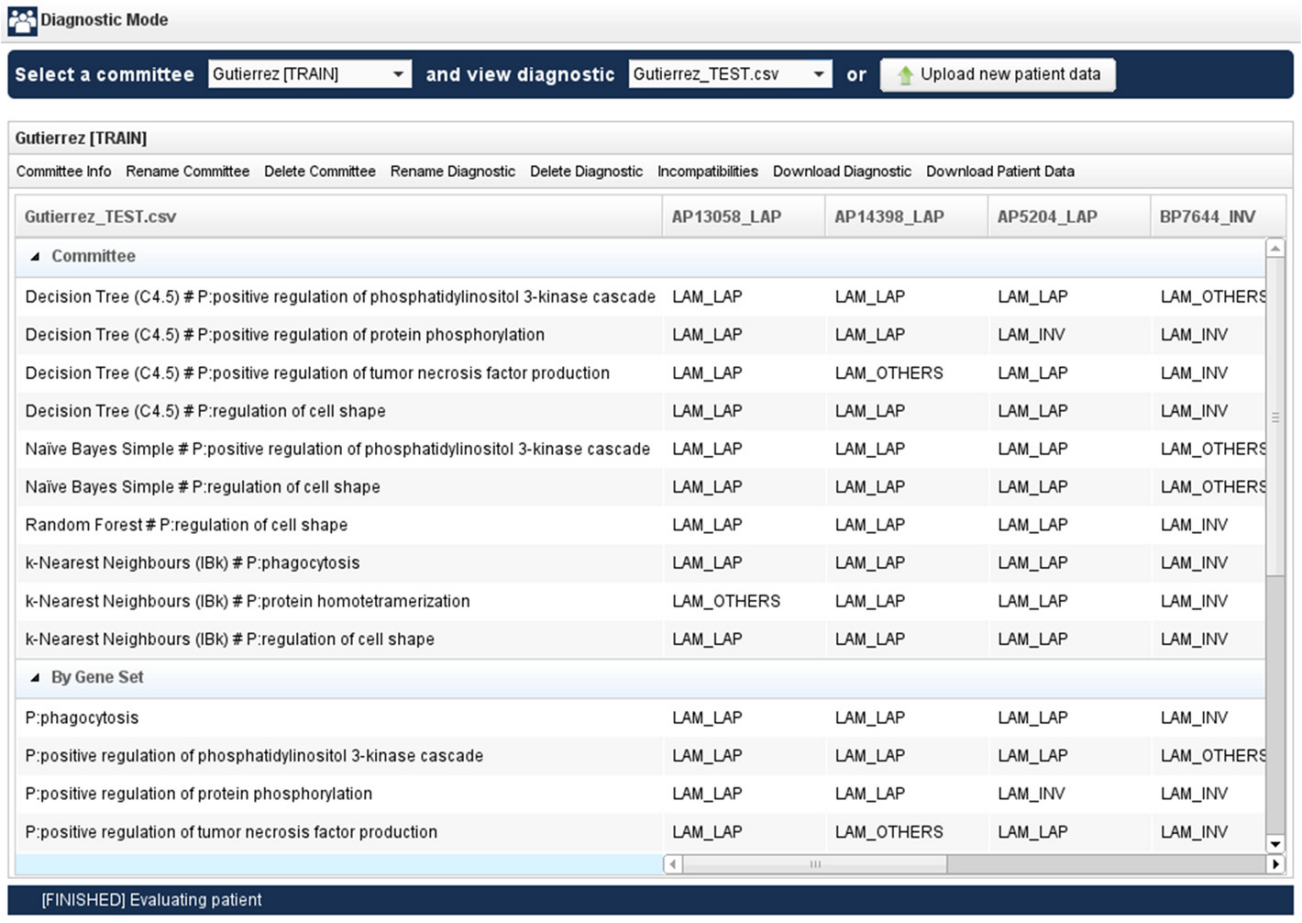
**Diagnostic view of geneCommittee application.** For each patient (columns) the diagnostic results are shown by expert and grouped by gene set, classifier and class.

Once the selected committee concludes the processing of the new dataset, geneCommittee presents the result of the classification process as showed in Figure 6.

The information displayed in Figure 6 is structured using a table where each column (except for the first) represents one different patient. In this table, rows are grouped in four main sections:

•Committee: each row contains the diagnostics of one member (expert) of the committee, that is to say, a classifier trained using only the biological information of its associated gene set. Committee members will select one single condition for each patient.

•By Gene Set: this section summarizes the committee member’s diagnostics by grouping the outputs of those experts that share the same gene set. Only the conditions with the highest number of votes are shown.

•By Classifier: in the same way as the previous area, this section groups the committee member’s diagnostics taking into consideration the type of the classifier.

•Voting: this section summarizes the whole diagnostic process carried out by showing the votes that each condition has received, along with a final row evidencing the condition or conditions with the higher number of votes.

The diagnostic view showed in Figure 6 also provides a helpful toolbar with several options for conveniently manage the information displayed.

### RNA-Seq support

The workflow implemented in our geneCommittee server is based on previous works [20,22] in which we defined successful knowledge-based classification protocols for analysing gene expression data. Although these approaches were initially intended to specifically handle DNA microarrays, the underlying workflows are generic and able to process other types of gene expression data sources. In this context, mainly motivated by the increasing importance and popularity of RNA-Seq techniques for measuring gene expression levels, in this work we also evaluate the suitability of our geneCommittee server for dealing with compatible RNA-Seq datasets.

## Results and discussion

In order to easily discover the main advantages of geneCommittee, a guest account was created with a publicly available dataset taken from previous work of Gutiérrez et al. [35]. Microarray data coming from this study (43 samples, 9188 genes and 4 distinct groups of acute myeloid leukemia, AML) was divided into two independent datasets for training (31 samples) and testing (12 samples) purposes. Table 1 presents the structure of the train and test files available in the help menu of our geneCommittee server.

**Table 1:** Structure of the train and test datasets available in geneCommittee

**Condition (disease)**	**Acronym**	**Training samples**	**Test samples**
Acute promyelocytic leukemia	LAP	7	3
AML with inversion 16	INV	3	1
Monocytic leukemias	M5	5	2
Other variants	OTH	16	6

By following the pipeline previously commented and implemented in our geneCommittee server, the first action to be done after the selection of the dataset (step 1 in Figure 3) is to choose the N most discriminative genes (in this example, N = 50 using Chi-square test). The next phase (step 3 in Figure 3) allows to automatically enrich the original microarray features with related biological knowledge regarding the set of genes obtained as output after the execution of the gene selection phase (step 2 in Figure 3). For this purpose, external information about biological processes, diseases, orthologous genes, and pathways, is dynamically gathered from GeneBrowser web services. In the generated view one can observe the association between the 5 base genes with 173 new concepts, such as the phagocytosis capacity, reported in studies about leukemia cells [36], and the regulation of actin cytoskeleton pathway [37]. By using this augmented knowledge, the user can easily select those concepts of interest for subsequently train different classifiers. With the goal of facilitating the selection of gene sets, the user can filter them by name, source, p-value or coverage of the gene set in the original dataset. In this case, we have filtered the resulting list to select those gene sets with a p-value < 0.0001 and with a minimum coverage of 25. The next phase of the workflow (step 4 in Figure 3) allows the selection and configuration of several classifiers to be initially trained with the available data and knowledge. By using the 5 available classification algorithms and the Kappa method, several threads are executed in parallel to obtain a list of 27×5 (135) kappa values (step 5 in Figure 3). The evaluation result can be easily inspected by the user with the goal of selecting those classifiers that best discriminate the train dataset. Note that some kappa values may display an ‘ERROR’ label, which means that the classifier could not be trained usually because the dataset contains a very low number of genes. In our example, 10 experts with highest kappa value were finally selected to compose the specific committee for AML disease (see Table 2):

**Table 2:** **Biologically interpretable classifiers ( *experts *) with their corresponding Kappa value**

**Id**	**Classifier**	**Biological concept**	**Kappa**
DT#1	Decision Tree (C4.5)	Positive regulation of phosphatidylinositol 3-kinase cascade	0.6687
DT#2	Decision Tree (C4.5	Positive regulation of protein phosphorylation	0.6511
DT#3	Decision Tree (C4.5	Positive regulation of tumor necrosis factor production	0.6347
DT#4	Decision Tree (C4.5)	Regulation of cell shape	0.6173
NBS#1	Naïve Bayes Simple	Positive regulation of phosphatidylinositol 3-kinase cascade	0.6146
NBS#2	Naïve Bayes Simple	Regulation of cell shape	0.6004
RF#1	Random Forest	Regulation of cell shape	0.6748
IBK#1	k-Nearest Neighbours (IBk)	Phagocytosis	0.6202
IBK#2	k-Nearest Neighbours (IBk)	Protein homotetramerization	0.6137
IBK#3	k-Nearest Neighbours (IBk)	Regulation of cell shape	0.8080

From the experts list comprising the final committee showed in Table 2, we can clearly observe the association of leukemia and specific biological processes such as positive regulation of phosphatidylinositol 3-kinase cascade [38] or the phagocytosis [36,39].

When working with geneCommittee it is recommended to include diversity among those gene sets and classifiers used. On one hand, adding different gene sets will expand the search field for geneCommittee to identify those sets containing the most discriminant genes. On the other hand, the variation in the classification schemes is important to get the best experts as, based on the ‘no free lunch theorem’, there is no single classification algorithm capable of classify every different case.

The final committee generated using the 6-step wizard for committee training can be later used in Diagnostic mode for performing posterior diagnoses of new cases. In our example, using the test dataset available in the help menu of geneCommittee, one can apply the previous AML committee to detect 12 other samples, upon the distinct experts can be combined through a majority voting schema to obtain a final classification. Table 3 summarizes the diagnosis output generated by geneCommittee. As it can be seen, using an unweighted majority vote among the 10 different experts, the committee was capable of correctly classifying each sample, although any single expert was not able to correctly classify all cases. This is a usual advantage of ensemble schemes in classification that we have already verified in our previous studies related with this work [20,22].

**Table 3:** Diagnosis output summary for the 12 samples taken from the Gutiérrez et al. dataset

	***AP13058***	***AP14398***	***AP5204***	***BP7644***	***CP13774***	***CP9949***	***XP12570***	***XP15833***	***XP170***	***XP17273***	***XP6209***	***XP9875***
DT#1	LAP	LAP	LAP	OTH	M5	M5	OTH	M5	OTH	LAP	INV	INV
DT#2	LAP	LAP	INV	INV	M5	M5	OTH	LAP	OTH	LAP	OTH	OTH
DT#3	LAP	OTH	LAP	INV	M5	M5	OTH	M5	OTH	OTH	LAP	OTH
DT#4	LAP	LAP	LAP	INV	M5	M5	M5	M5	OTH	LAP	LAP	OTH
NBS#1	LAP	LAP	LAP	OTH	OTH	M5	OTH	OTH	OTH	OTH	OTH	OTH
NBS#2	LAP	LAP	LAP	OTH	OTH	M5	OTH	OTH	OTH	OTH	OTH	OTH
RF#1	LAP	LAP	LAP	INV	M5	M5	M5	OTH	OTH	OTH	OTH	OTH
IBK#1	LAP	LAP	LAP	INV	M5	M5	M5	LAP	OTH	OTH	INV	M5
IBK#2	OTH	LAP	LAP	INV	INV	M5	OTH	OTH	OTH	OTH	OTH	OTH
IBK#3	LAP	LAP	LAP	INV	M5	M5	INV	OTH	INV	OTH	OTH	OTH
INV	0	0	1	**7**	1	0	1	0	1	0	2	1
M5	0	0	0	0	**7**	**10**	3	3	0	0	0	1
LAP	**9**	**9**	**9**	0	0	0	0	2	0	3	2	0
OTH	1	1	0	3	2	0	**6**	**5**	**9**	**7**	**6**	**8**
**Condition**	LAP	LAP	LAP	INV	M5	M5	OTH	OTH	OTH	OTH	OTH	OTH

Most voted condition for each sample is highlighted with bold text.

The diagnostic information provided by geneCommittee is one of the most important features of our tool. As each classifier is related with one known biological concept, classification results are easier to understand for non-expert users and, additionally, further conclusions related with these concepts can be extracted. Moreover, the detailed voting for each sample can be used as a simple confidence measurement.

Complementarily, a similar study was designed with our geneCommittee server but using RNA-Seq datasets from the portal of The Cancer Genome Atlas [40]. For this purpose, a data matrix was created with samples related to breast invasive carcinoma (BRCA) disease. Available samples were filtered with the goal of only selecting RNASeqV2 data coming from the IlluminaHiSeq_RNASeqV2 platform of the University of North Carolina (UNC).

As a result, two datasets were created containing normalized gene expression information of the data captured. The first dataset, intended to use for committee training, was composed by 115 samples of the TCGA’s batches 93 and 96. The second dataset, intended to use for testing, was composed by 112 samples of the TCGA’s batches 103 and 105. In both cases, the number of available genes is 20502 with two conditions (i.e.: tumor and normal). These datasets are publicly available in the help menu of our geneCommittee server.

Using our geneCommittee server, a diagnostic committee was created with the first dataset using the following straightforward configuration: (*i*) electing the 300 best genes according to the Chi-square test, (*ii*) selecting those sets obtained in the enrichment step for guaranteeing a p-value < 0.0001 and a minimum coverage of 150 and (*iii*) choosing those classifiers (trained with default parameters) with a kappa value > = 0.95 in the evaluation step. This resulted in a final committee composed of 34 experts.

By using the second dataset for testing purposes, the diagnostic performance of the previously generated committee was remarkable: from the 112 testing samples, and using the unweighted majority vote approach, only two samples were misclassified and one of them with a tie between the two classes.

Currently, our geneCommittee server has been successfully tested in Internet Explorer 8, Firefox 16, Opera 9.62 and Safari 3 browsers working on Windows XP/Vista/7, Ubuntu Linux 10.04 version and Mac OSX 10.5 of Intel architecture.

## Conclusion

Nowadays, expression microarrays represent a sound technology for disease diagnosis and prognosis. However, its full exploitation depends greatly on the success of statistical approaches that are able to extract accurate evidences from raw data. Another major challenge in the area is to provide end-user tools that allow them to test hypothesis and to make diagnostics in a seamless environment that hides the complexity of statistical software packages. This paper introduces geneCommittee, a web based application that: a) integrates a set of well-known classification methods in an ensemble framework; b) enables the enrichment of original dataset variables with biologically relevant features; c) provides training classification solutions that allow to select classifiers based on the biological relevance and/or discriminative power; and d) permits applying the experts, a mix of classifier/variable, to diagnose new patients.

In our work, geneCommittee was tested using two different gene expression datasets (i.e.: DNA microarray and RNA-Seq). In both cases, our server has demonstrated a good performance which encourages its suitability for analysing data coming from different gene expression data sources.

The main innovation of geneCommittee is related with its ability to combine the advantages of using multiple complementary classifications algorithms with the benefits obtained from the true integration of biological knowledge in the classification process. From this perspective, our geneCommittee server deals with two main prerequisites needed for the development of valuable translational microarray-based diagnostic tools: (*i*) a solid understanding of the relative strengths and weaknesses of underlying classification methods and (*ii*) a biologically plausible and understandable behaviour of such models from a biological point of view.

## Availability and requirements

**Project name:** geneCommittee

**Project home page**: http://sing.ei.uvigo.es/GC/

**Project source page**: https://github.com/michada/GeneCommittee

**Operating system**: Platform independent

**Programming language**: Java

**Other requirements**: Internet browser: Explorer 8, Firefox 3, Opera 9.62 and Safari 3

**License**: GNU GPL

## Competing interests

The authors declare that they have no competing interests.

## Authors’ contributions

All authors contributed equally to conception of geneCommittee. MR-J and JA programmed the final solution. JLO provided use cases and tested the usability of the software. FFR generated geneCommittee documentation. JLO and FFR wrote the paper while MR-J and JPA provided comments and discussion. All authors read and approved the final manuscript.

## Supplementary Material

Additional file 1 **Expert manual.** This document will guide the user through a step-by-step tutorial showing the capabilities of geneCommittee for extensively testing the discriminatory power of biologically relevant gene sets in microarray data classification. Click here for file
