# Supplementary material for: geneCommittee: a web-based tool for extensively testing the discriminatory power of biologically relevant gene sets in microarray data classification
Source: BMC Bioinformatics. 2014 Jan 30;15:31. doi: 10.1186/1471-2105-15-31 (PMC3909759; doi:10.1186/1471-2105-15-31)
Supplement: Additional file 1 — Expert manual. This document will guide the user through a step-by-step tutorial showing the capabilities of geneCommittee for extensively testing the discriminatory power of biologically relevant gene sets in microarray data classification. [file 1471-2105-15-31-S1.pdf]

GENECOMMITTEE

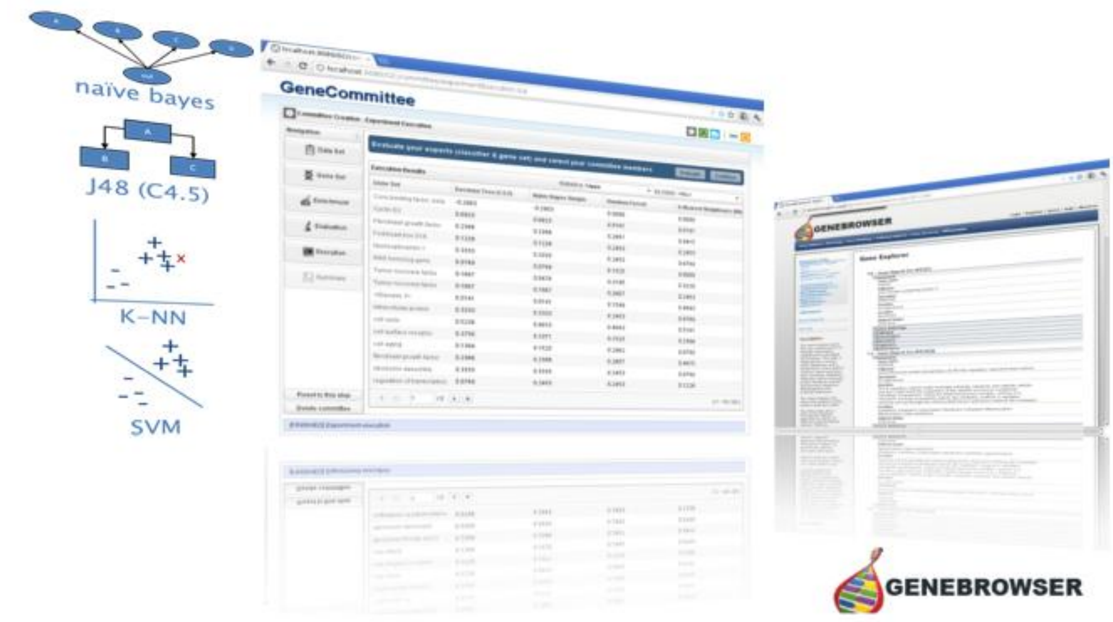

http://sing.ei.uvigo.es/GC/

Expert Manual

This document will guide you through a step-by-step tutorial showing the capabilities of GENECOMMITTEE for extensively testing the discriminatory power of biologically relevant gene sets in microarray data classification.

Contents

About the tool 1
Login 2
Welcome to GENECOMMITTEE 3
Committee Training 4
Data Set Selection 4
Gene Selection 5
Enriched Gene Set 6
Evaluation Configuration 8
Experiment Execution 9
Committee Summary 10
Diagnostic Mode 12
Data Management 15
Data Set Format 16

About the tool

GENECOMMITTEE is a web-based interactive tool giving specific support to study of the discriminative classification power of custom hypothesis in the form of biologically relevant gene sets. With a straightforward and intuitive interface, GENECOMMITTEE is able to provide valuable information for diagnostic analyses and clinical management decisions based on systematically evaluating custom hypothesis over different data sets using complementary classifiers, a key aspect in clinical research.

GENECOMMITTEE's main features:

- *Upload, store and manage different microarray data files.*
- *Several configurable classification techniques, including Naïve Bayes, Decision Trees (C4.5/J48), K-NN (K-nearest neighbours) and SVM (Support Vector Machines).*
- *User-friendly 6-step wizard to create new committees.*
- *Classifier evaluations are executed in parallel in our server.*
- *Email notifications, containing a direct link to the results stored in the server.*

GeneBrowser integration:
GENECOMMITTEE application is interconnected with the successful GeneBrowser server, a web-based tool for gene set enrichment.

Login

On the welcome page the user can choose to either login using a demo account or create a new account. Using the demo account (the fields are already fulfilled), the user will have access to several data sets and committees previously created.

To create a new account the login form available on the home page can be used. The account information must be based on the user's email address, which will be used to send login information, and processing results, when completed. Each registered user will have an independent workspace inside the GENECOMMITTEE server.

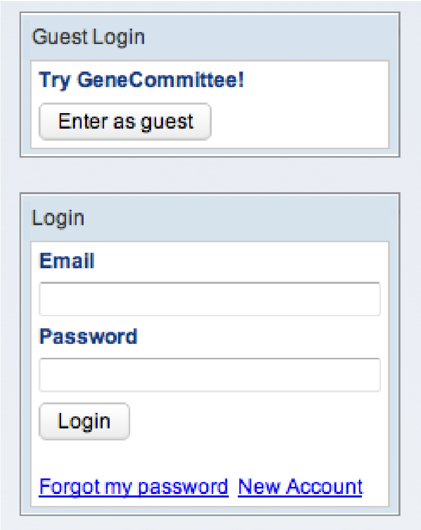

An email message is sent to the user after filling in all the fields in the Login form in order to validate the registration.

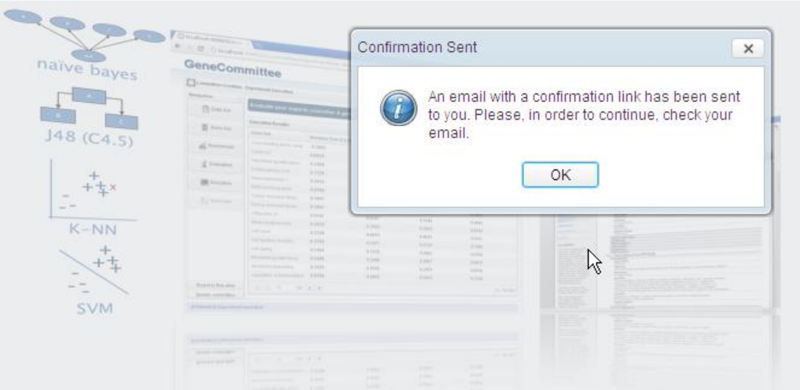

Welcome to GENECOMMITTEE

Once logged in, the user has access to the home page of GENECOMMITTEE, and is able to start a new work or continue a previously started project.

The email address of the active user is shown at the top of the page. Quick-navigation buttons can be found there, linking to the Help, user Personal Data, Committee Training, Diagnostic Mode, Data Management, Home and Logout pages.

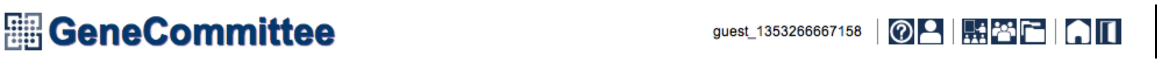

The user must change his/her Email Notifications setting in the Personal Data tab in order to receive an email message after task completion. Note that email notifications will be sent to the active user, so you should create and use your own account to receive them.

GENECOMMITTEE is divided in three sub-tools: (*i*) Committee Training, (*ii*) Diagnostic Mode, and (*iii*) Data Management, as shown below.

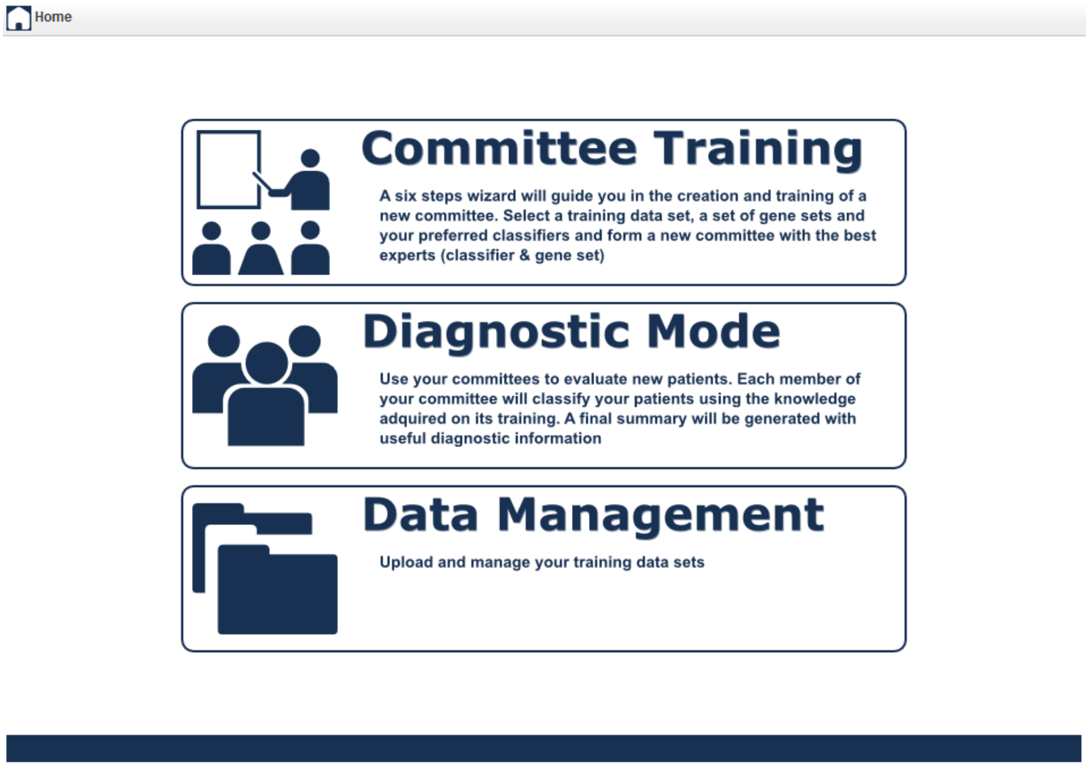

Prior to training any committees the user is required to upload the desired data sets using the Data Management screen. All uploaded data sets are stored in GENECOMMITTEE, allowing the user to suspend a project and continue it later without risk of losing previous work. Note that the demo account provides two default data sets for tool testing purposes. Once the data sets are uploaded, the user can directly proceed to the Committee Training tool and perform the desired experiments. All tasks presented in this guide were performed using the demo account and the ‹Valk› default data set.

Committee Training

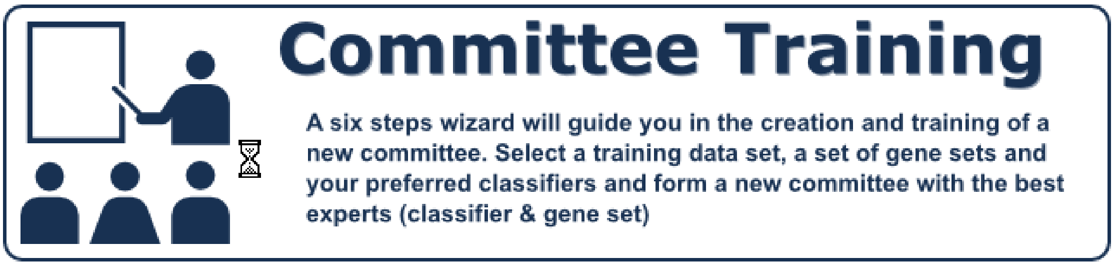

The Committee Training wizard follows a workflow whose mains steps are presented on the left toolbar.

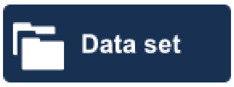	***Data Set Selection***

The first step consists of selecting the desired data set. To do so, click on the "Select data set" dropdown menu, choose the previously uploaded data set and click "Continue".

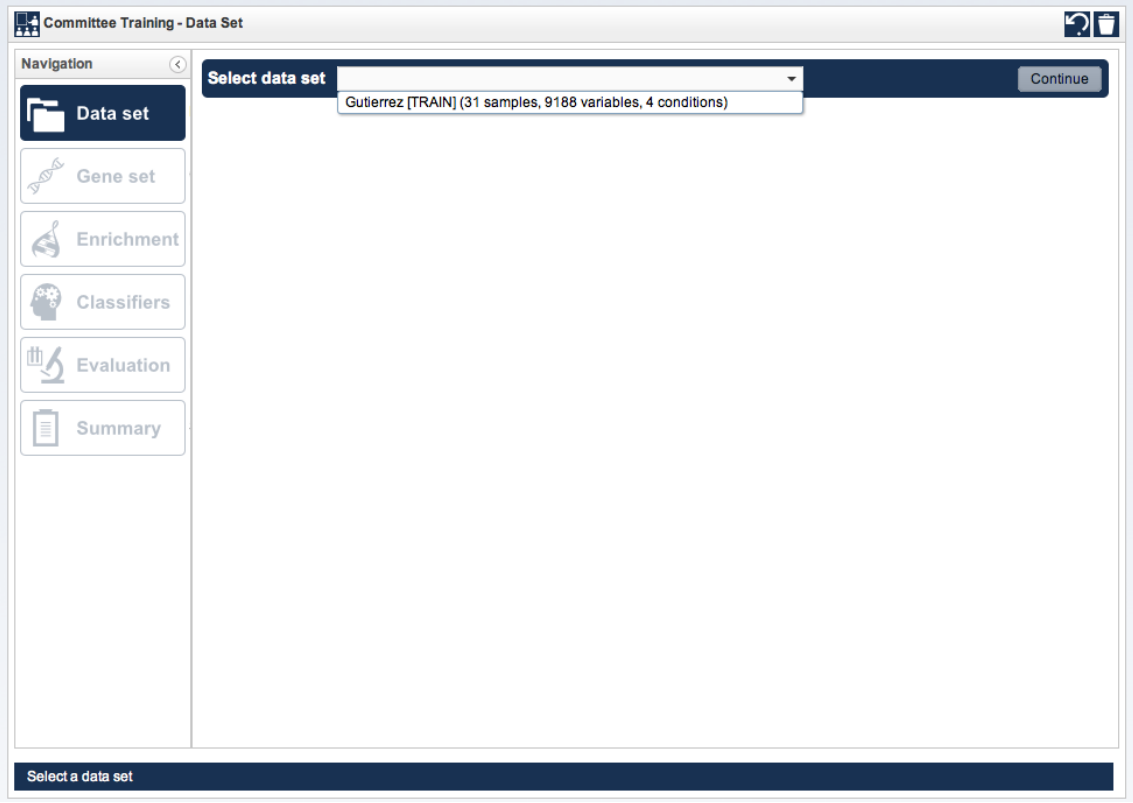

Note that if the data set is changed during committee training, and before the whole workflow is completed and saved, the training process already performed upon the current dataset will be lost. Please make sure you select the correct data set, before proceeding.

For each data set, general information is shown relating to conditions and samples. Specific information properties can be expanded or collapsed. The information is presented in tabular view and is grouped in two tables: (*i*) Metadata (upper table), consisting of the sample size, number and discrimination of the conditions, and the number of genes present, and; (*ii*) Conditions (lower table), where each discriminated condition is represented.

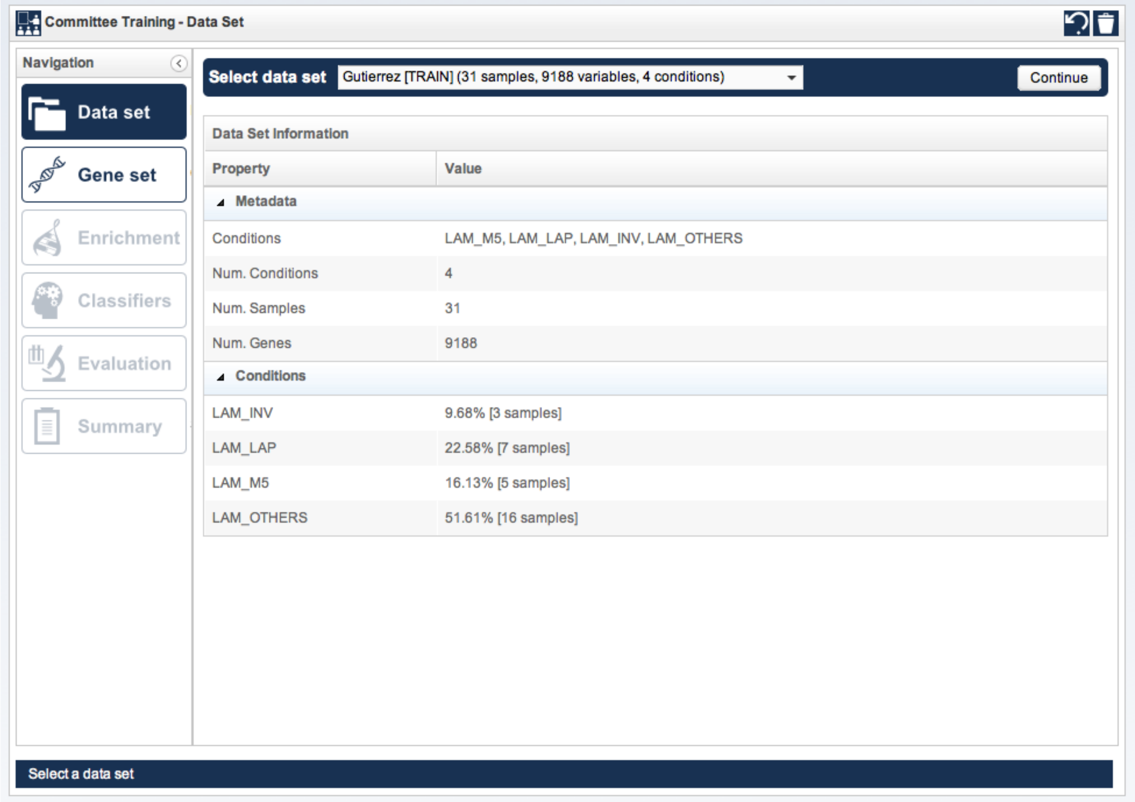

The selected data set can then undergo gene selection.

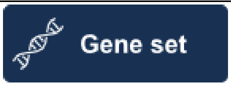

Gene Selection

Using the dropdown menus in the blue bar shown below it is possible to filter the number of genes with higher discriminative ranking. The available options for gene selection are chi-squared distribution, information gain split method, gain ratio, and the relief-f feature filtering algorithm. Additionally, the user can opt to binarize numeric attributes and/or merge missing values in order to better adapt raw data to the selected filtering algorithm.

Once all the details are configured, it is necessary to press the “Select Genes” button to automatically perform the gene selection process.

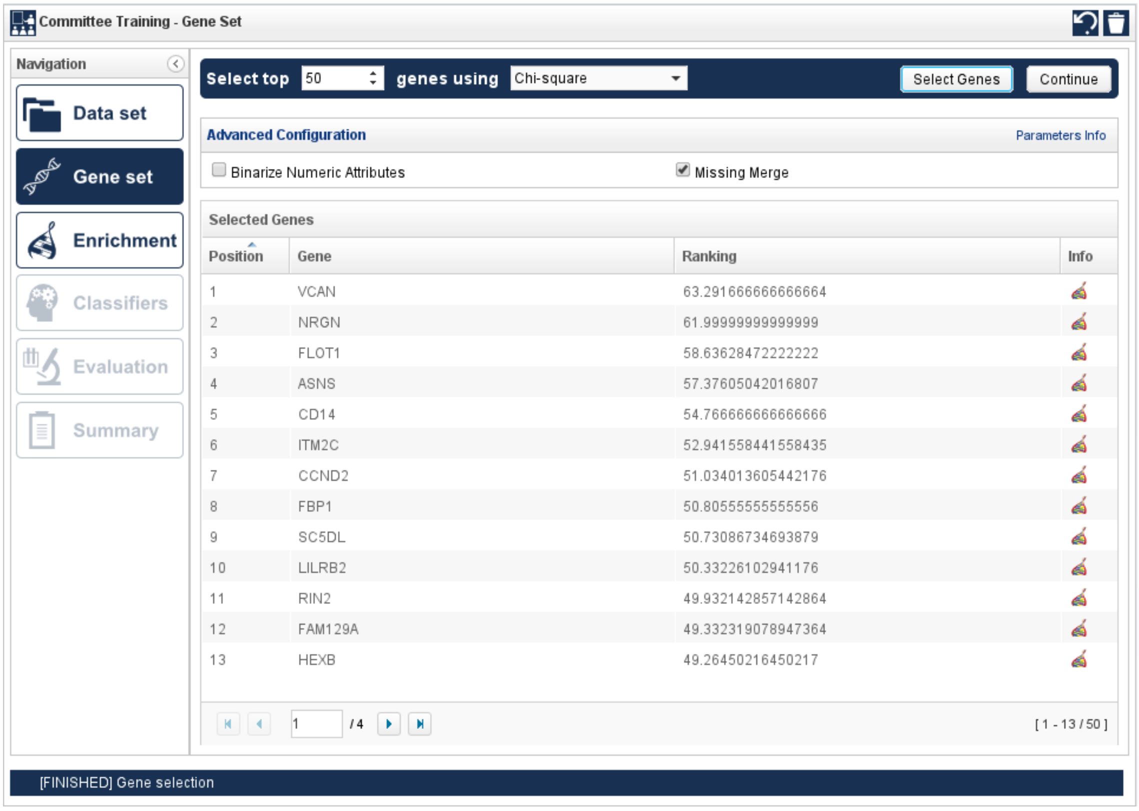

In the above example, five genes were selected using the information gain split method. Genes are ordered by test ranking.

In the following step of the workflow, the selected genes can be enriched using the integrated GeneBrowser tool.

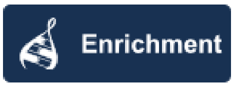

Enriched Gene Set

The selected genes are transported to the Enrichment screen. Clicking the “Enrich” button results in a query to GeneBrowser and returns information regarding each gene. As soon as gene enrichment is completed, a new table containing the retrieved data is immediately shown.

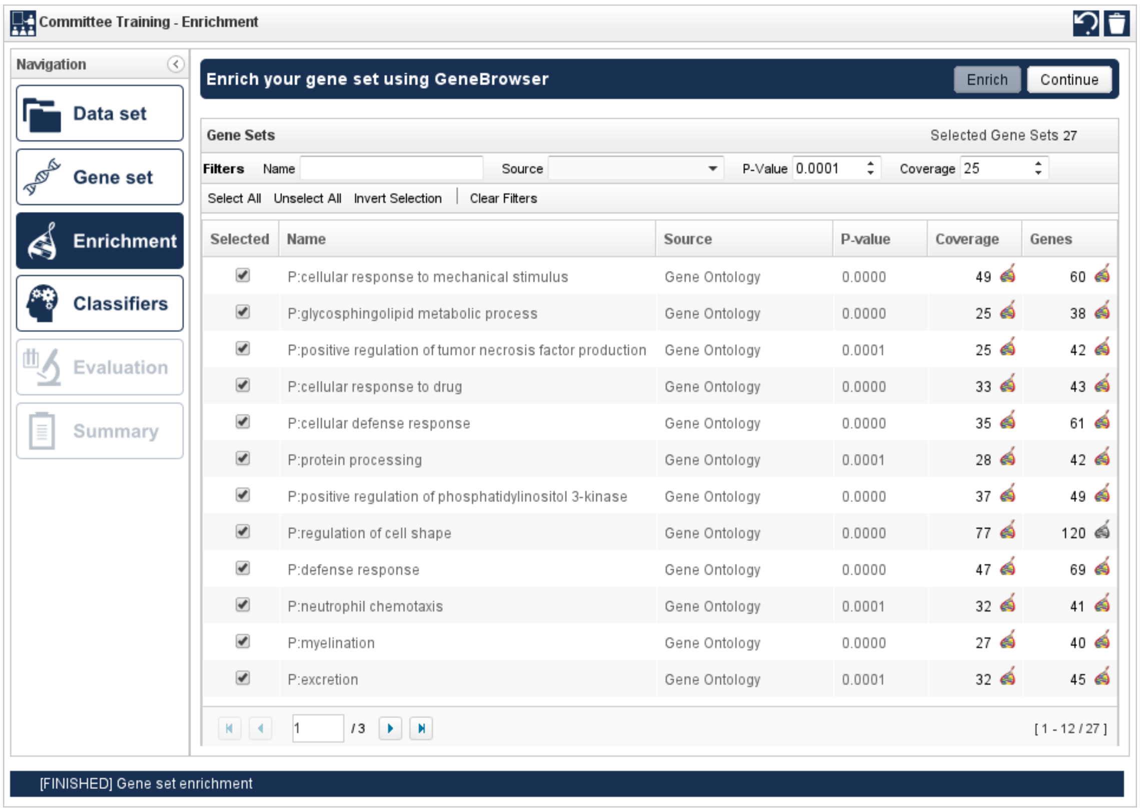

All entries are selected by default, but the user has the possibility of choosing the desired enriched gene sets. Unwanted entries can be deselected using the checkbox in the first column.

The previous table contains the name of the found items, their sources, the associated *p*-value, the number of genes involved and the respective link to GeneBrowser[1]. This link allows the user to obtain detailed information for entries of interest without leaving GENECOMMITTEE.

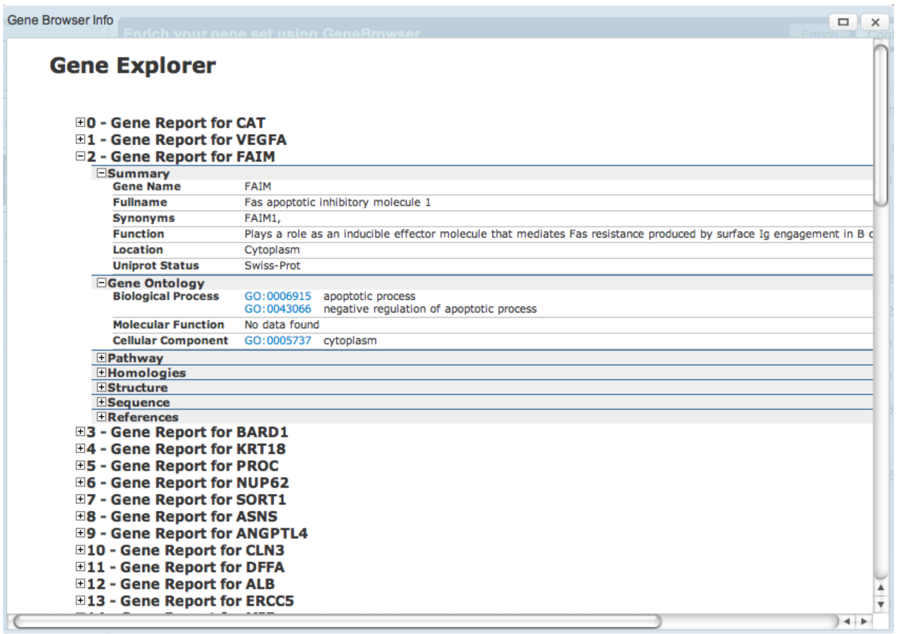

[1] http://bioinformatics.ua.pt/gb2

Following gene set enrichment, it is necessary to configure the classifiers and the evaluation strategy.

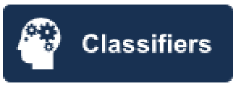

Evaluation Configuration

The evaluation system in GENECOMMITTEE is very flexible, allowing the use of a single classifier or multiple classifier combinations. All classifiers have their advantages and drawbacks, so it is important to assign the most appropriate classifier to the data set in use. Our idea of committee allows choosing the best combination of methods for a specific data set.

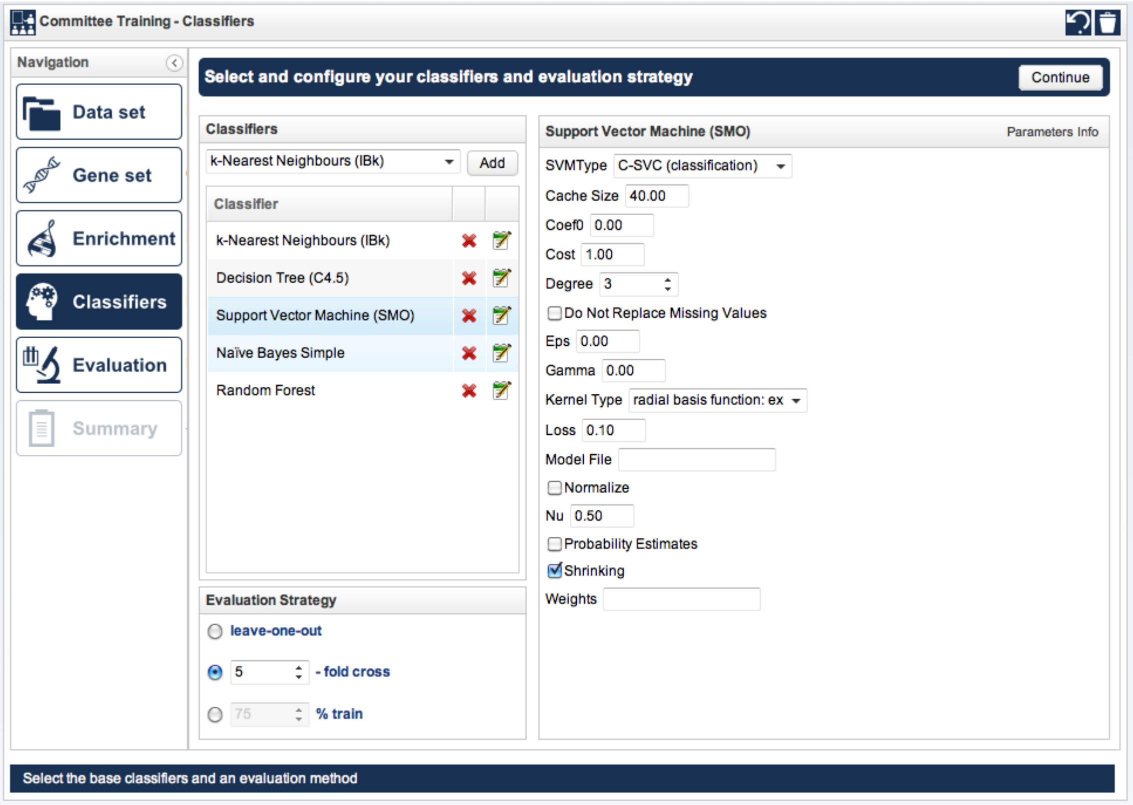

There are five classifiers available: (*i*) k-nearest neighbours, (*ii*) decision trees, (*iii*) support vector machine, (*iv*) naïve Bayes, and (*v*) random forest. After being added, each classifier can be refined to perfectly assess the input data. This feature can be accessed by clicking the “Edit” button at the right of the classifier, just next to the “Delete” button. Also, the names of the classifiers can be changed for easier identification.

The last task consists of setting the evaluation strategy to finally perform the experiment. Note that clicking the “Continue” button will not promptly initiate the job, but will take the user to the Evaluation screen.

Evaluation | *Experiment Execution*

Once all the settings are defined, clicking the “Evaluate” button initiates the task. An attractive feature of GENECOMMITTEE is live-visualization of the experiment execution. If any error is detected, the user can cancel the active task clicking the “Abort” button in the progress bar.

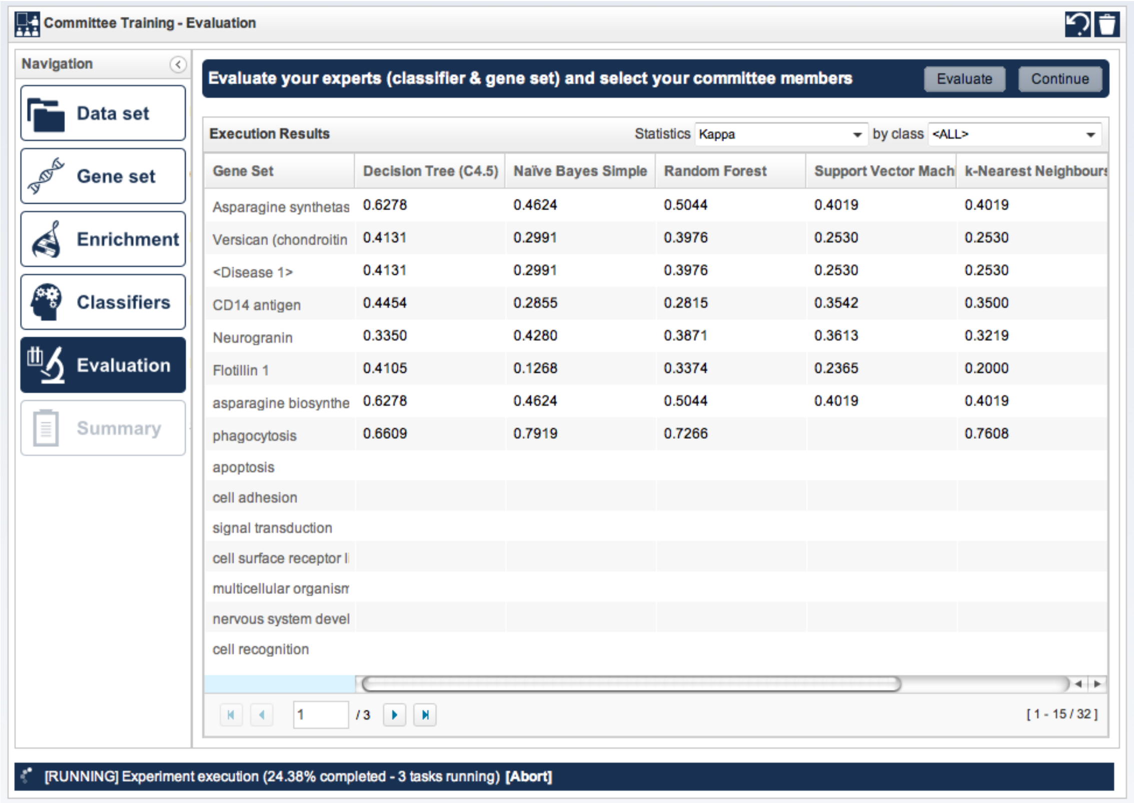

As soon as the job is finished the user must pick the desired experts (classifier and gene features) for building a new committee. In order to evaluate the performance of each expert, the statistical analysis of the execution results can be adjusted. Allowed statistics include Cohen’s Kappa, accuracy, precision, recall, specificity and F-measure. This allows better perception of the results' significance.

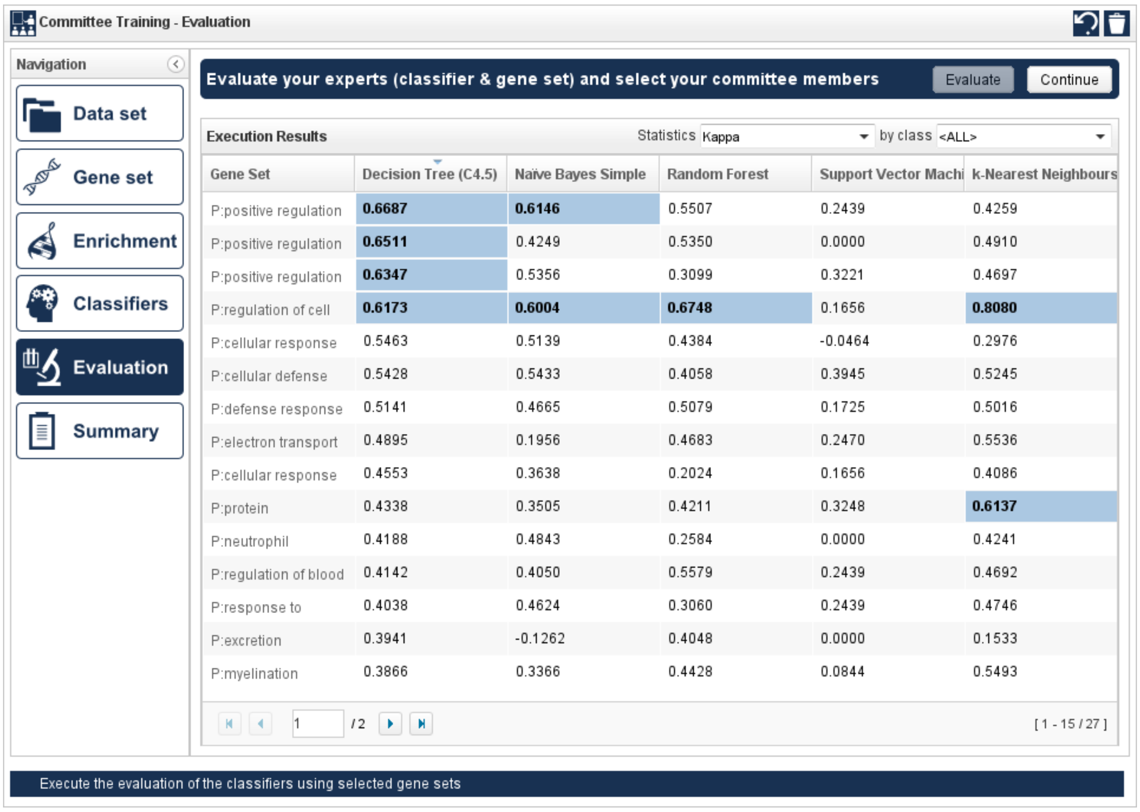

Another interesting feature is the possibility of visualizing the results of a specific class, or in the case shown, a condition. Note that results of all classes are shown by default. Statistic types and class visualization can be changed using the dropdown menus above the results table.

Once the experts are finally selected, the user must then save the information about the newly created committee.

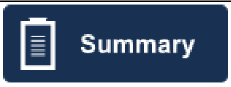

Committee Summary

Finally, the Committee Summary screen shows general information about the input data set, how gene selection was performed and committee details. The newly created committee can be instantly used in the Diagnostic Mode to evaluate new patients.

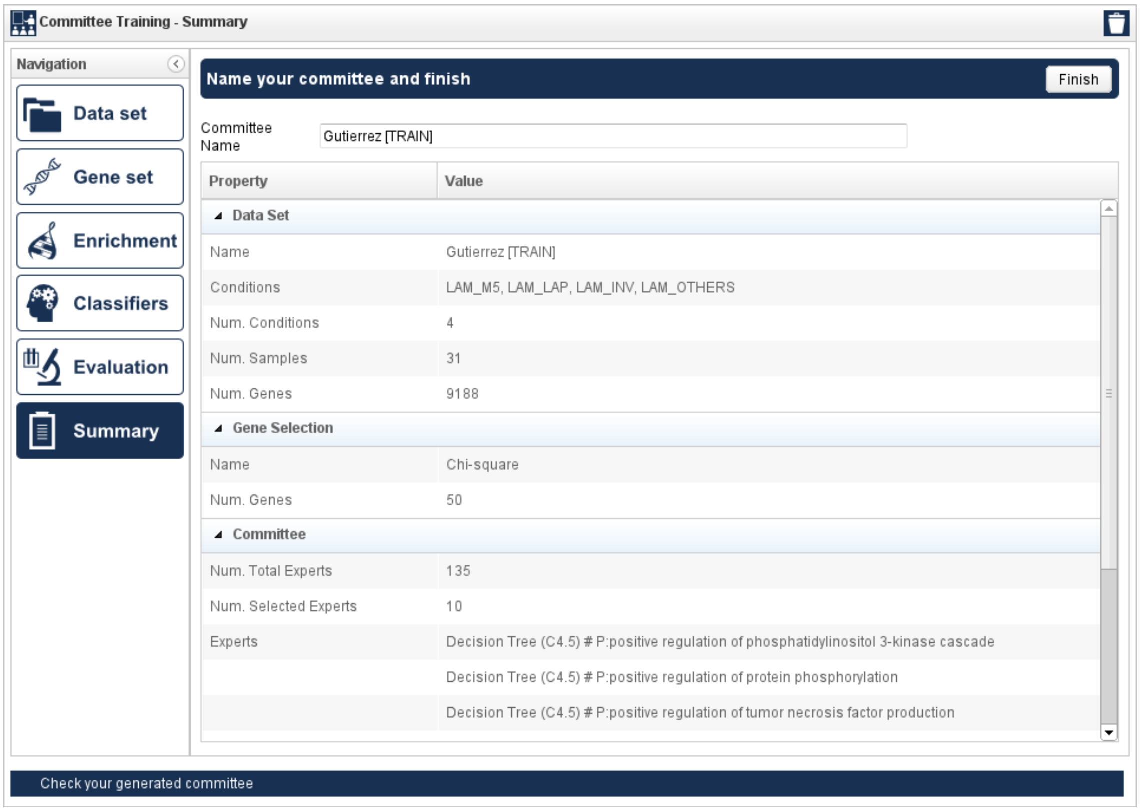
Committee Training - Summary
Navigation
Data set
Gene set
Enrichment
Classifiers
Evaluation
Summary
Name your committee and finish
Finish
Committee Name
Gutierrez [TRAIN]
Property
Value
Data Set
Name
Gutierrez [TRAIN]
Conditions
LAM_M5, LAM_LAP, LAM_INV, LAM_OTHERS
Num. Conditions
4
Num. Samples
31
Num. Genes
9188
Gene Selection
Name
Chi-square
Num. Genes
50
Committee
Num. Total Experts
135
Num. Selected Experts
10
Experts
Decision Tree (C4.5) # P:positive regulation of phosphatidylinositol 3-kinase cascade
Decision Tree (C4.5) # P:positive regulation of protein phosphorylation
Decision Tree (C4.5) # P:positive regulation of tumor necrosis factor production
Check your generated committee

Diagnostic Mode

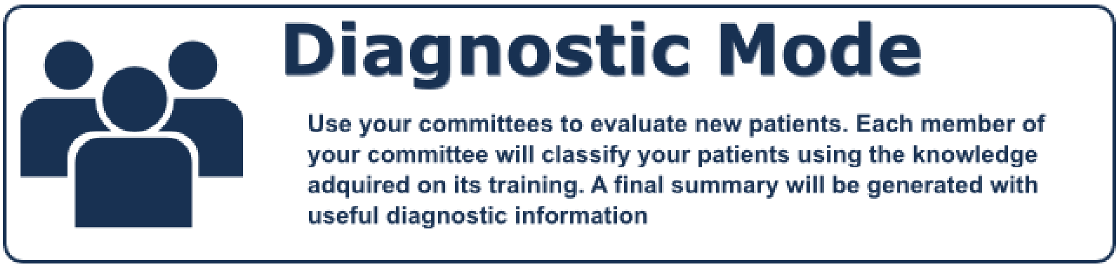

In Diagnostic Mode the user can apply the created and trained committees to evaluate new patients. The carefully selected experts are compared to the test (patient) data to identify probable new disease cases.

Firstly, it is necessary to select the newly created committee using the dropdown menu in the blue bar. Then, patient data must be uploaded using the button on the right of the same bar.

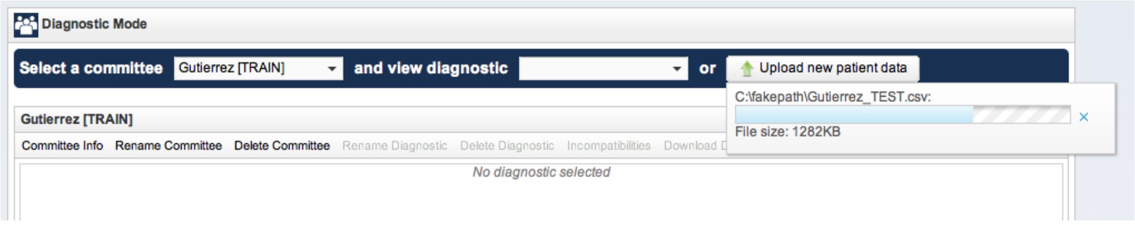

The first time a user uses a committee for diagnostic, he/she should upload the patient data to be evaluated. Using the “Upload new patient data” button, the user can upload a data file with the patient data. This file follows the same format as described in the “Data Set Format” subsection but in this case, the “CLASS” row should not contain any value (it is recommended to use the ‘?’ symbol as value).

Example patient data file:

UNIQID	NAME	GSM20962.CEL	GSM20701.CEL	GSM20702.CEL
#	CLASS	?	?	?
1	HDAC1	0.26299268	0.45148313	-10.665.518
2	MAPRE1	0.16962652	-0.6115107	-0.5892962
3	NEO1	-0.80309725	-11.346.912	-12.663.207

As soon as the user uploads the patient data, the committee will start working on the diagnostic of the patients. The status bar at the bottom of the window shows you the diagnostic progress.

An information window will notify that the work has finished. When the diagnostic is complete, the user can explore the results by selecting the diagnostic in the second combo box of the upper tool bar. The diagnostic loading may take a while so please be patient while GENECOMMITTEE loads it.

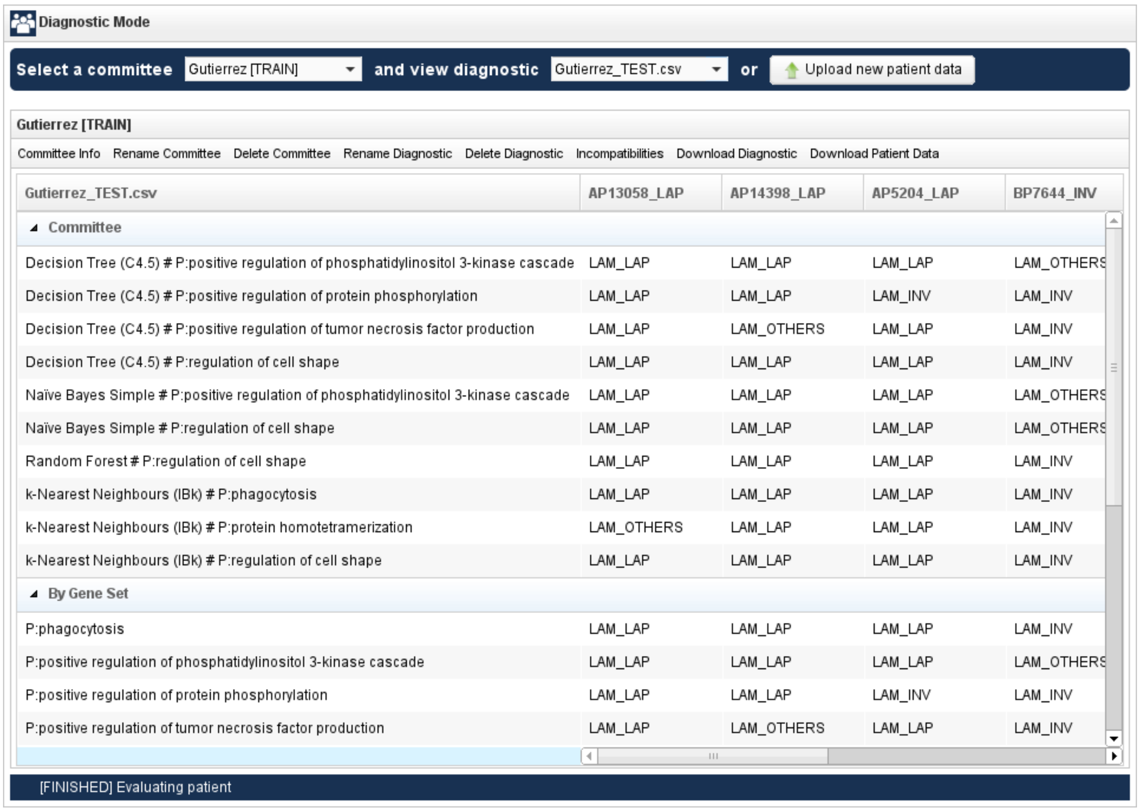

Diagnostic results are presented in a table where each column (except the first) represents one patient. In this table, the rows are grouped in four main sections:

- **Committee**: Each row contains the diagnostics of one member of the committee, a classifier trained using only the gene information of its associated gene set. Committee members will select one single condition for each patient.
- **By Gene Set**: This section summarizes the committee member's diagnostics by grouping the outputs of those members that share the same gene set. Only the condition or conditions with the highest number of votes are shown.
- **By Classifier**: In the same way as the previous section, this section groups the committee member's diagnostics by the classifier type employed.
- **Voting**: This final section summarizes the whole diagnostic process by showing the votes that each condition has received, along with a final row that shows the condition or conditions with the highest number of votes among all the committee members.

The diagnostic view also provides a helpful toolbar with several options that will help you to manage your trained committees and diagnostics. The options included in this toolbar are the following:

- **Committee Info**: Shows a popup window with information about the current committee. The figure below shows an example of this information panel.
- **Rename Committee**: This option allows you to rename the current committee.
- **Delete Committee**: This option allows you to delete the current committee. You must take into account that deleting a committee will provoke the deletion of the associated diagnostics.

- **Rename Diagnostic**: This option allows you to rename the current diagnostic. Diagnostics will be named by default with the name of the uploaded patient data set file.
- **Delete Diagnostic**: This option allows you to delete the current diagnostic.
- **Download Diagnostic**: With this option you can download the diagnostic information as a CSV file.
- **Download Patient Data**: This option allows you to download the original patient data set file.

Data Management

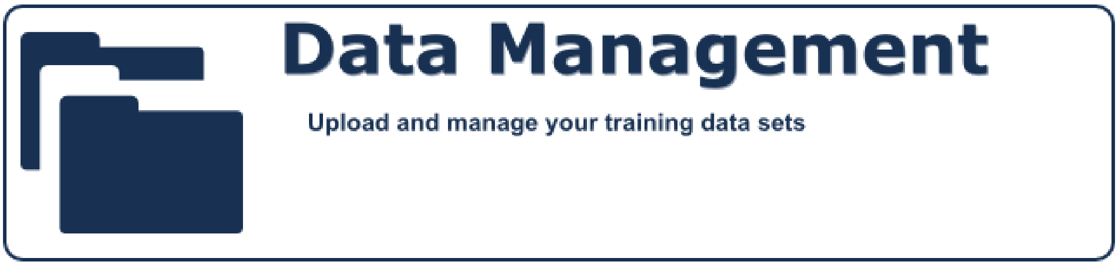

In Data Management the user can upload and manage the data sets that will be used for committee training. As can be seen in the following figure, the Data Management view is divided into two main panels.

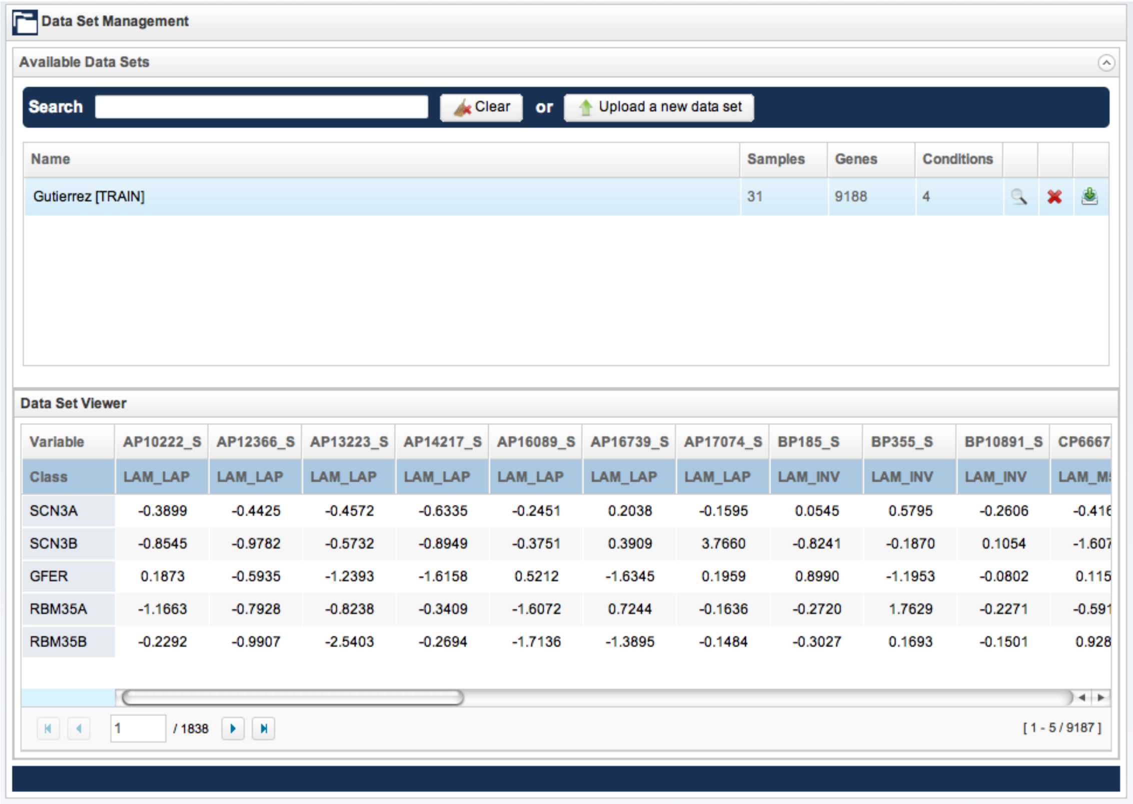

The upper panel lists the existing data sets and allows the user to manage them. Using the tool included in the top toolbar, the user can search through the existing data sets or upload a new data set. Below this toolbar, a list of data sets shows the main features of each and allows the user to visualize, delete or download each data set.

When the user chooses to visualize a data set, it will be shown in the bottom panel. The data set will be displayed in a table view where the gene ids are listed in the first column, while the following columns contain the sample data. The first row contains the sample ids, the second row contains the class (condition) of each sample and the following rows contain the expression level of corresponding gene. In order to facilitate data set visualization, the upper panel can be collapsed (as shown in the following figure) using the top right button.

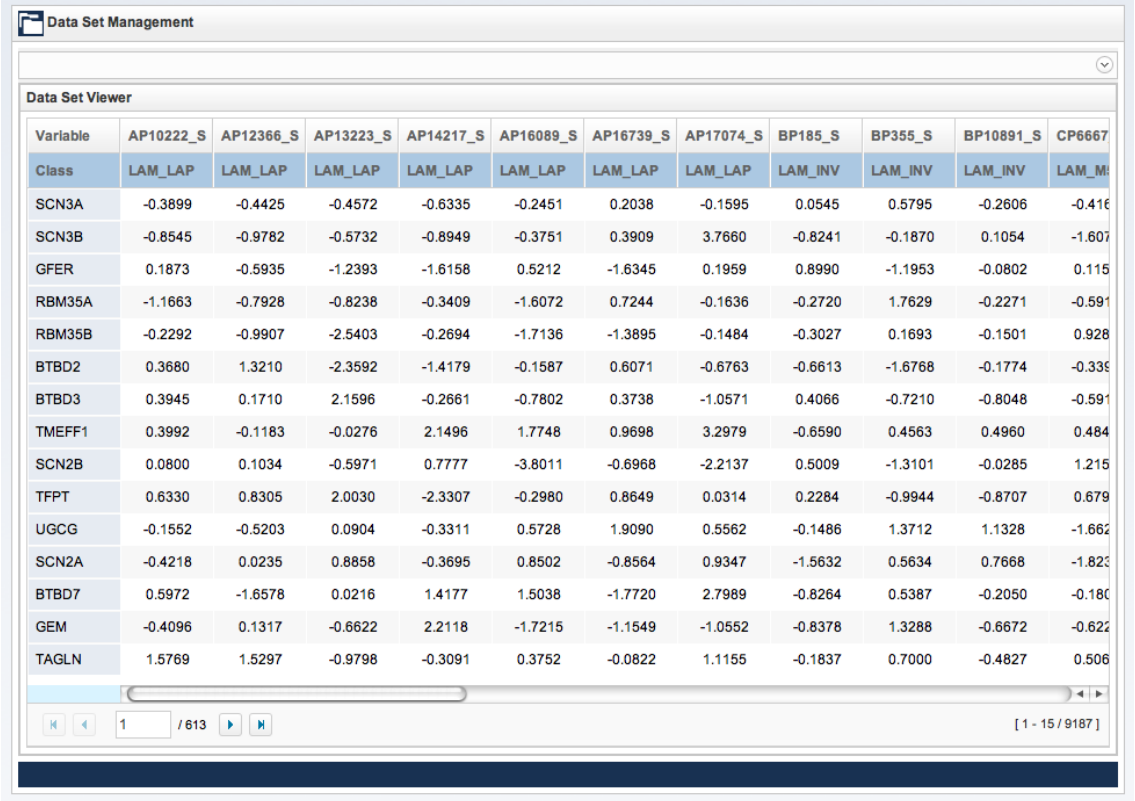

Data Set Management

Data Set Viewer

Variable	AP10222_S	AP12366_S	AP13223_S	AP14217_S	AP16089_S	AP16739_S	AP17074_S	BP185_S	BP355_S	BP10891_S	CP6667
Class	LAM_LAP	LAM_LAP	LAM_LAP	LAM_LAP	LAM_LAP	LAM_LAP	LAM_LAP	LAM_INV	LAM_INV	LAM_INV	LAM_M
SCN3A	-0.3899	-0.4425	-0.4572	-0.6335	-0.2451	0.2038	-0.1595	0.0545	0.5795	-0.2606	-0.416
SCN3B	-0.8545	-0.9782	-0.5732	-0.8949	-0.3751	0.3909	3.7660	-0.8241	-0.1870	0.1054	-1.607
GFER	0.1873	-0.5935	-1.2393	-1.6158	0.5212	-1.6345	0.1959	0.8990	-1.1953	-0.0802	0.115
RBM35A	-1.1663	-0.7928	-0.8238	-0.3409	-1.6072	0.7244	-0.1636	-0.2720	1.7629	-0.2271	-0.591
RBM35B	-0.2292	-0.9907	-2.5403	-0.2694	-1.7136	-1.3895	-0.1484	-0.3027	0.1693	-0.1501	0.928
BTBD2	0.3680	1.3210	-2.3592	-1.4179	-0.1587	0.6071	-0.6763	-0.6613	-1.6768	-0.1774	-0.339
BTBD3	0.3945	0.1710	2.1596	-0.2661	-0.7802	0.3738	-1.0571	0.4066	-0.7210	-0.8048	-0.591
TMEFF1	0.3992	-0.1183	-0.0276	2.1496	1.7748	0.9698	3.2979	-0.6590	0.4563	0.4960	0.484
SCN2B	0.0800	0.1034	-0.5971	0.7777	-3.8011	-0.6968	-2.2137	0.5009	-1.3101	-0.0285	1.215
TFPT	0.6330	0.8305	2.0030	-2.3307	-0.2980	0.8649	0.0314	0.2284	-0.9944	-0.8707	0.679
UGCG	-0.1552	-0.5203	0.0904	-0.3311	0.5728	1.9090	0.5562	-0.1486	1.3712	1.1328	-1.662
SCN2A	-0.4218	0.0235	0.8858	-0.3695	0.8502	-0.8564	0.9347	-1.5632	0.5634	0.7668	-1.823
BTBD7	0.5972	-1.6578	0.0216	1.4177	1.5038	-1.7720	2.7989	-0.8264	0.5387	-0.2050	-0.180
GEM	-0.4096	0.1317	-0.6622	2.2118	-1.7215	-1.1549	-1.0552	-0.8378	1.3288	-0.6672	-0.622
TAGLN	1.5769	1.5297	-0.9798	-0.3091	0.3752	-0.0822	1.1155	-0.1837	0.7000	-0.4827	0.506

1 / 613 [1 - 15 / 9187]

Data Set Format

GENECOMMITTEE accepts comma separated value (CSV) files with the following structure:

- Samples are represented in the columns
- Genes are represented in the rows

Therefore, each sample (column) contains a:

- Sample ID;
- Class.

Each gene (row) contains a:

- Numerical identifier; Note that this gene identifier is not a database identifier, only a unique value to identify the gene in the data set;
- Name.

Each cell in the samples x genes matrix consists of the expression value of each gene.

These characteristics will require a specific input file format. The first row contains:

- First column: “UNQID”;
- Second column: “NAME”;
- Other columns: sample identifiers.

The second row contains:

- First column: “#”;

- Second column: "CLASS";
- Other columns: class names.

Other rows contain:

- First column: a numerical value different in all lines to identify each gene;
- Second column: gene names;
- Other columns: gene expression values (decimal, separated by a dot) for each sample, one column per sample.

Example file:

UNIQID	NAME	GSM20962.CEL	GSM20701.CEL	GSM20702.CEL
#	CLASS	LAM_RESTO	LAM_INV	LAM_INV
1	HDAC1	0.26299268	0.45148313	-10.665.518
2	MAPRE1	0.16962652	-0.6115107	-0.5892962
3	NEO1	-0.80309725	-11.346.912	-12.663.207
